# Assessment of the Possibility of Reducing Energy Consumption and Environmental Pollution in the Steel Wire Manufacturing Process

**DOI:** 10.3390/ma16051940

**Published:** 2023-02-26

**Authors:** Maciej Suliga, Radosław Wartacz, Joanna Kostrzewa, Marek Hawryluk

**Affiliations:** 1 Faculty of Production Engineering and Materials Technology, Czestochowa University of Technology, 19 Armii Krajowej Av., 42-201 Czestochowa, Poland; 2 Independent Researcher, 1b Mykanowska Str., 42-240 Kościelec, Poland; 3 The Jacob of Paradies University in Gorzów Wielkopolski, Teatralna 25 Str., 66-400 Gorzów Wielkopolski, Poland; 4 Department of Metal Forming and Metrology, Wroclaw University of Science and Technology, 5 Lukasiewicza Str., 50-371 Wrocław, Poland

**Keywords:** steel, wire drawing, dies, energy consumption, CO_2_ emission

## Abstract

This paper describes research on the influence the technology of zinc-coated steel wire manufacturing has on the energy and force parameters of the drawing process, energy consumption and zinc expenditure. In the theoretical part of the paper, the theoretical work and drawing power were calculated. Calculations of the electric energy consumption have shown that usage of the optimal wire drawing technology results in a 37% drop in energy consumption, which in the course of a single year translates to savings equal to 13 TJ. This, in turn, results in the decrease of CO_2_ emissions by tons and a total decrease of the eco-costs by approximately EUR 0.5 mln. Drawing technology also influences the losses of the zinc coating and CO_2_ emissions. Properly adjusted parameters of the wire drawing technology allow obtaining a zinc-coating that is 100% thicker, translating to 265 tons of zinc, whose production generates 900 tons of CO_2_ and incurs eco-costs equal to EUR 0.6 mln. Optimal parameters for drawing, from the perspective of decreased CO_2_ emissions during the zinc-coated steel wire manufacturing, are as follows: usage of the hydrodynamic drawing dies, angle of the die reducing zone α = 5°, and drawing speed of 15 m/s.

## 1. Introduction

Steel and steel products are the primary construction material in many industries. This results from large deposits of iron ores worldwide and low costs of iron manufacturing compared with alternative construction materials [1,2]. With a possible exception of small fluctuation in demand and other factors, such as the COVID-19 pandemic, the dynamic development of many economies results in a continuous increase of steel production that currently exceeds 1500 million tons annually (e.g., according to World Steel Association, it was 145.7 million tons in October 2021). On the other hand, steel production negatively impacts on the natural environment and contributes to the CO_2_ emissions and thus increases the greenhouse effect [3,4,5,6].

Energy used in the steel manufacturing processes is primarily produced in coal power plants that are also a source of gases and dust [7,8]. This is why it is a priority to decrease energy consumption and, in consequence, reduce the environmental impact as well [9,10].

Metal products manufacturing is a multi-stage process that can generally be broken into three constituent parts. The first stage is the production of raw iron and subsequent steel processes [9], the second stage is mould or continuous casting [10], resulting in a semi-product such as a billet of a given cross-section (square, rectangular, circular) and length, which is a material for the third stage, i.e., plastic shaping in cold and hot processes (forging, extruding, rolling, drawing, stamping) [11]. A substantial part of metal products consists of wire products such as springs [12,13], ropes [14,15], nets, chains, and connecting elements (bolts, screws, rivets, nails) [16]. Depending on the size, one factory can produce between a few and 500 thousand tons of wire and wire products annually. This means that the contribution of the metal drawing industry to environmental pollution is substantial.

The charge material used in wire manufacturing is hot rolled steel, especially low-carbon steel containing between 0.05 and 0.95%C [17,18]. The drawing process is complex and consists of many technological operations that include an anti-corrosive metallic coating of both the charge material and manufactured wire (coating with zinc, tin, brass, bronze and copper) [19,20], thermal processing (recrystallization annealing for low-carbon steels, patenting for high-carbon steels) [21,22], surface processing (scale removal, application of lubricant carriers), and drawing operations [23].

Because of ecology, environmental protection, and environmental tariffs charged by governmental institutions, this industry branch is undergoing substantial development resulting in successive adoption of environment-friendly technologies by the factories [24]. For example, hot processes used in steel patenting began to replace molten-lead baths with fluidal deposits [25]. Surface processing replaces chemical processes, and the removal of scale from the surface of the rolled steel through the application of hydrochloric and sulfuric acids is being phased out by mechanical methods, such as bending the rolled steel in rollers with subsequent additional cleaning with canvas or sandpaper [26,27]. As for lubricants and lubricant carriers, usage of borax and its derivatives is either limited or, in the case of some countries, including EU member states, completely banned.

Reduction of energy consumption and emissions in the process of steel wire manufacturing can be achieved through optimization of the drawing process. The main parameters of the process that heavily influence the force parameters of the process and consequently also on energy consumption include the type and shape of a drawing die (the tool where the material is plastically deformed) [28,29,30,31,32,33] and drawing speed [34,35,36].

Currently, due to the reduction of the costs of the manufacturing of wire and wire products, drawing processes use multi-stage drawing machines, allowing the drawing of the wire in more than a dozen subsequent drafts, with the speeds reaching 30 m/s in the last draft (drawing of non-coated high-carbon steel wires). Maximum drawing speed depends on the drawing machine, total compression size, steel type, corrosion-resistant metallic coating type, drawing method, die geometry (including drawing angle), and lubrication conditions [37,38]. These parameters influence the deformation resistance, wire temperature [39,40], friction conditions in the wire/die contact zone [41], and total energy consumption [42].

In drawing hard steel wires with zinc coating, the soft metallic coating is subjected to grinding and fracturing, which results in the decrease of the zinc quantity remaining on the wires, negatively impacting the corrosion resistance of the manufactured wire. A thicker layer of zinc is initially applied to offset the impact and to account for the losses during the drawing process. Current processes of zinc production via metallurgical methods, as well as the methods of applying zinc coating on the rolled steel and wires, contribute to substantial pollution, several times higher than steel.

Considering all factors mentioned above, it seems reasonable for the manufacturing plants to take actions aimed at reducing energy consumption and greenhouse gas emissions.

The aim of this paper is to analyze the possibilities of reducing energy consumption, zinc losses, and pollution generated by the metal drawing industry related to the manufacturing of zinc-coated steel wires.

## 2. Materials and Methods

### 2.1. Manufacturing Process of Hot-Dip Galvanized Steel Wires

**Figure 1:**
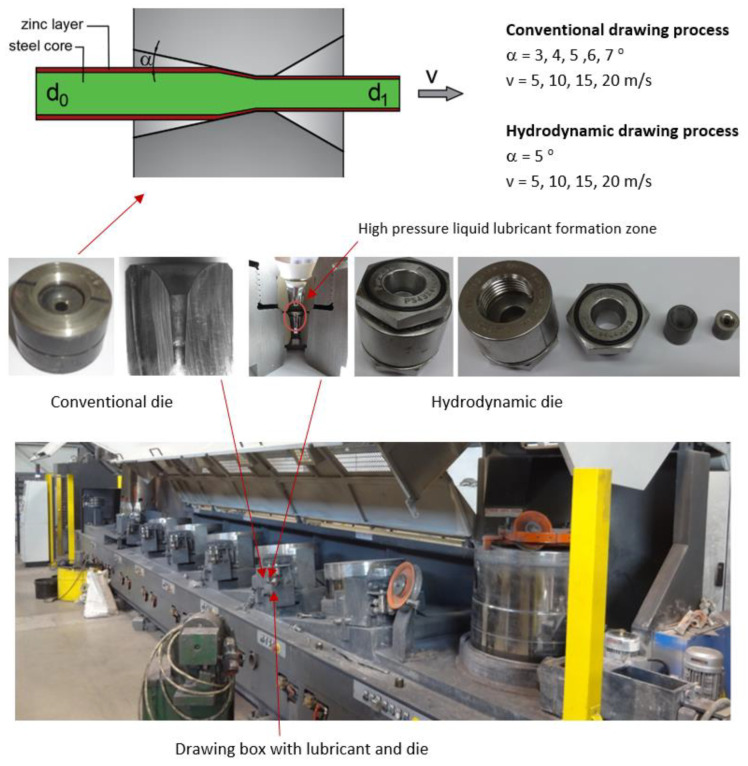
Model and parameters of the zinc-coated steel wire drawing process using a Mario Frigerio multi-stage drawing machine, where α—the angle of the die compression zone, v—drawing speed, d_0_, d_1_—initial and end diameter of the wire.

Analysis of the opportunities to reduce the energy consumption in the steel wire manufacturing process was based on theoretical research and experimental wire drawing in industrial conditions and using existing, commonly utilized tools and machinery. Experimental research was conducted on a steel wire manufacturing line utilizing a modern Mario Frigerio multi-stage wire-drawing machine. The charge material was 5.5 mm rolled C42D steel (0.42% C), zinc-coated in a hot-dip galvanization process. The resulting product was formed in seven drafts, eventually resulting in 2.2 mm wire, using conventional and hydrodynamic dies (the latter allows for fluid friction and reduction of the friction coefficient in the drawing process). The model and parameters of the drawing process are presented in Figure 1.

### 2.2. Methodology

Analysis of the possibility of reducing energy consumption in the wire drawing process has been based on theoretical research and experimental wire drawing process in actual industrial conditions.

Parameters of the multi-stage drawing process and resulting energy consumption depend on many factors, including die geometry, drawing method, and drawing speed. Given these factors, this paper established the influence of drawing technology on drawing power.

Analysis of the energy and force parameters of the drawing process can be conducted based on the available empirical equations, computer simulations, and experimental measurements made in industrial conditions.

Theoretical drawing power N_ct_ has been calculated using Marcol’s Equation (1) of the drawing work [43], namely:

(1) $$W_{c}=V\cdot R_{m_{śr}}\cdot ln\frac{A_{0}}{A_{1}}\left(1+\frac{\mu }{\alpha }+\frac{2\alpha }{3ln\frac{A_{0}}{A_{1}}}\right)$$

where:

W_c_—total drawing work, J·10^−^^3^,

V—the volume of the wire drawn in the time t = 1 s, mm^3^,

R_m_śr__—average value of the wire strength before and after, MPa,

A_0_, A_1_—initial and end wire diameter, mm,

μ—friction coefficient,

α—half of the angle of the die compression cone, rad.

Drawing work and drawing power are related by following the equation below:

W = N · t

where: N—drawing power, t—time.

In the calculation of the drawing work, the assumed time is t = 1 s, thus:

N_ct_ = W_c_/1 [kW].

The calculations assume a friction coefficient μ = 0.07 (conventional dies) and μ = 0.03 (hydrodynamic dies) [23]. The calculations were made for the drawn wire, in seven drafts, in the conical dies with the drawing angle equal to 3, 4, 5, 6, and 7°, with the drawing speed of 5, 10, 15 and 20 m/s. The tensile strength of wire on the subsequent drawing stages was, respectively: 790, 920, 992, 1055, 1107, 1165, and 1275 MPa.

Theoretical research allowed us to calculate theoretical drawing work and drawing power. Subsequently, to verify the obtained results, a power consumption measurement has been conducted in a real multi-stage wire drawing process in actual industrial conditions. Industrial research included the measurement of the total instantaneous drawing power required to implement the entire drawing process using the power consumption reading field of the Mario Frigerio machine. To perform a complete analysis, the percentage of engine load on the respective drawing stages has been read from the control panel of the drawing machine.

Following the industrial research, calculations were made to estimate the energy required to manufacture 100,000 tons of zinc-covered wire, which corresponds to an annual output of a large wire factory. Idemat software (app version 2.9.0.) has been used to analyze the influence of the consumption of electric energy produced by a coal-burning power plant on environmental pollution and associated eco-costs. According to the data obtained from the Idemat software, the total eco-costs for producing 100 MJ from a coal-fired power plant are EUR 4.35. On the other hand, producing 100 MJ of energy from a coal-fired power plant emits 29.91 kg of CO_2_. The next step was to calculate carbon emissions and eco-costs for the production of 100,000 tons of wires. For all tested variants of drawing, based on the actual measured energy consumption in the drawing process, the value of CO_2_ emissions and eco-costs was calculated. 

Subsequently, using the weighing method, the amount of zinc coating in the drawing process has been calculated, allowing the determination of losses of zinc depending on the drawing technology (relative to 100,000 tons of wire per year). Zinc production and hot-dip zinc-coating process affect the natural environment more severely than steel production. This is why it was essential to determine the influence of zinc losses on the increase in the environmental pollution level. Then, knowing the total amount of zinc coating the wires and the total annual factory output (100,000 of coated wire), an analysis of the zinc losses, CO_2_ emissions, and eco-costs was conducted. Much like in the case of energy consumption calculation, Idemat software has been used (app version 2.9.0.).

## 3. Theoretical and Experimental Analysis of the Energy Consumption in the Steel Wire Manufacturing Process

**Figure 2:**
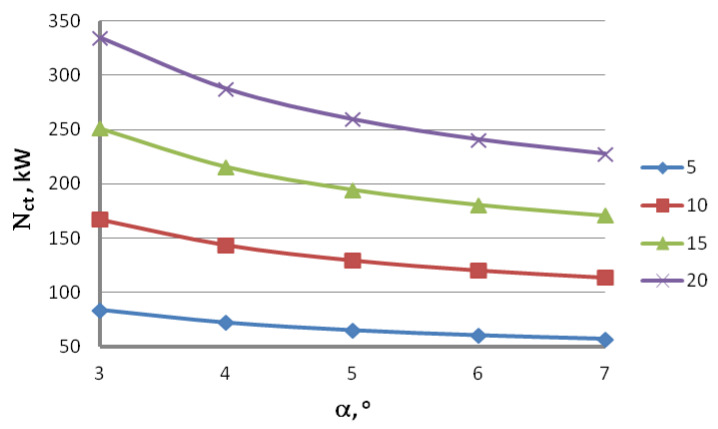
Influence of the drawing angle on the total drawing power N_ct_ of the wire with an initial diameter of 5.5 mm and end diameter of 2.2 mm for wires drawn with speed v = 5, 10, 15, 20 m/s.

**Figure 3:**
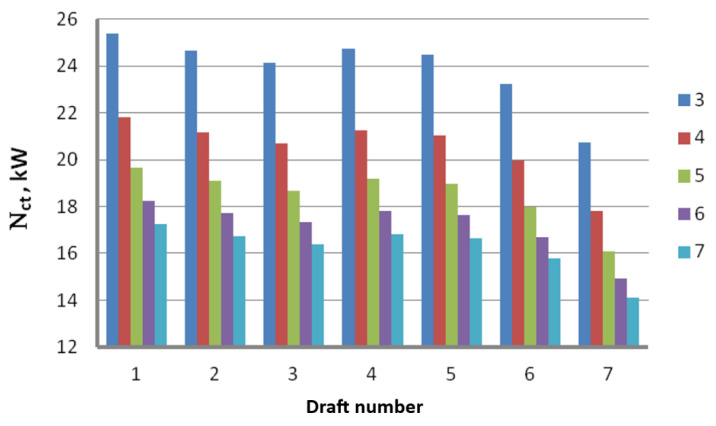
Influence of the drawing angle α on the theoretical wire drawing power in respective drafts; v = 10 m/s.

Theoretical analysis of the drawing process based on Marcol’s equation allowed us to create graphs demonstrating the influence of the drawing speed, die geometry and drawing method on the theoretical drawing power. Figure 2 presents the total (including seven drafts) theoretical drawing power, while Figure 3 illustrates the theoretical drawing power in respective drafts.

Based on the data presented in Figure 2 and Figure 3, we can state that the drawing angle significantly influences the power and force parameters of the steel wire drawing process, with the larger drawing angle translating to lower drawing power values. Theoretical research allows us to say that the theoretical drawing power increases proportionally to the drawing speed. It has been proven that the wire drawing in dies at an angle α = 7° results in a drawing power that is 32% lower than drawing in dies at an angle α = 3°. Higher drawing power values resulting from drawing at smaller values of α angle can be linked to a larger contact area between the wire and the die, which causes the drawing tension to increase, which in turn translates to higher drawing power. Higher force parameters for this variant suggest that in the drawing process for the zinc-coated wires, small drawing angles might result in the increased temperature of the outer layer of the wire. This is corroborated by the preliminary research conducted by the authors of this paper [44]. They demonstrated that during the drawing of uncoated wires, an increase of the drawing angle from 3° to 7° results in a ~30% decrease in the temperature on the wire surface. Thus, lower α angle values in the zinc-coated wire drawing process can contribute to the removal of the outer layer of zinc coating in a die while additionally causing the coating to warm up. The higher temperature of the outer layer of the wire, especially in the case of a drawing speed equal to 20 m/s, increases the propensity of the zinc coating to stick to the die and drawing drums. 

**Figure 4:**
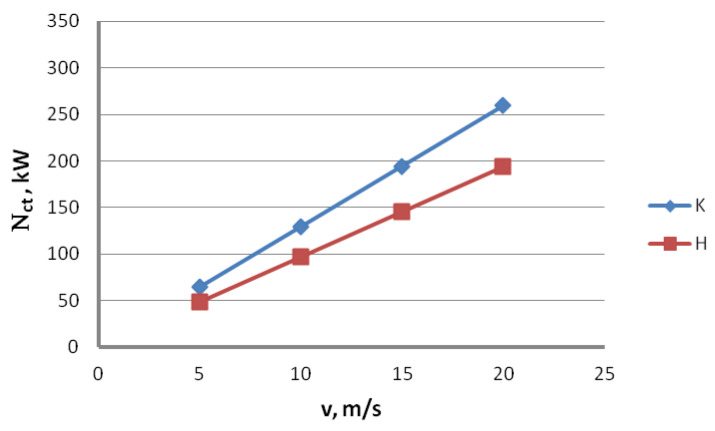
Influence of the drawing speed v on the total drawing power in conventional dies (K) and hydrodynamic dies (H).

**Figure 5:**
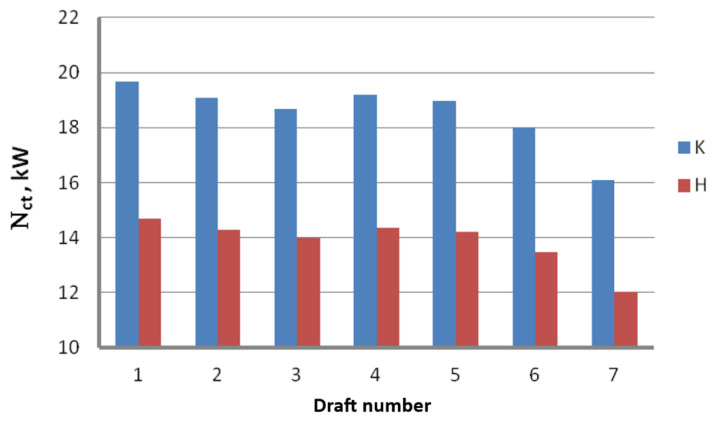
Theoretical drawing power in respective drafts for the wire drawn in conventional dies (K) and hydrodynamic dies (H); v = 10 m/s.

The subject literature demonstrates that using hydrodynamic dies is a known method of reducing friction and temperature of the drawn wire. This is why this paper compares the total drawing power in the wire drawing process using conventional dies and hydrodynamic dies related to the function of drawing speed and the drawing powers in respective drafts for a drawing speed of v = 10 m/s. The results are presented in Figure 4 and Figure 5.

Figure 4 and Figure 5 allow us to say that the drawing method significantly influences the drawing power. Utilization of the hydrodynamic dies in the multi-stage steel wire drawing process causes the drawing power to drop by approximately 25%, regardless of the drawing speed. Thus, using the hydrodynamic dies in the zinc-coated wire drawing process should benefit the drawing conditions (better lubrication and lower wire temperature). Consequently, wires drawn with hydrodynamic dies should also have a thicker and more homogenous zinc coating.

However, that the theoretical calculations presented above are based on “perfect” drawing conditions, where the coefficient of friction between the material and tool is constant, even though in the real drawing process, the friction and lubrication conditions differ between drafts. This is why the determination of the drawing power in industrial conditions seems to be prudent.

**Figure 6:**
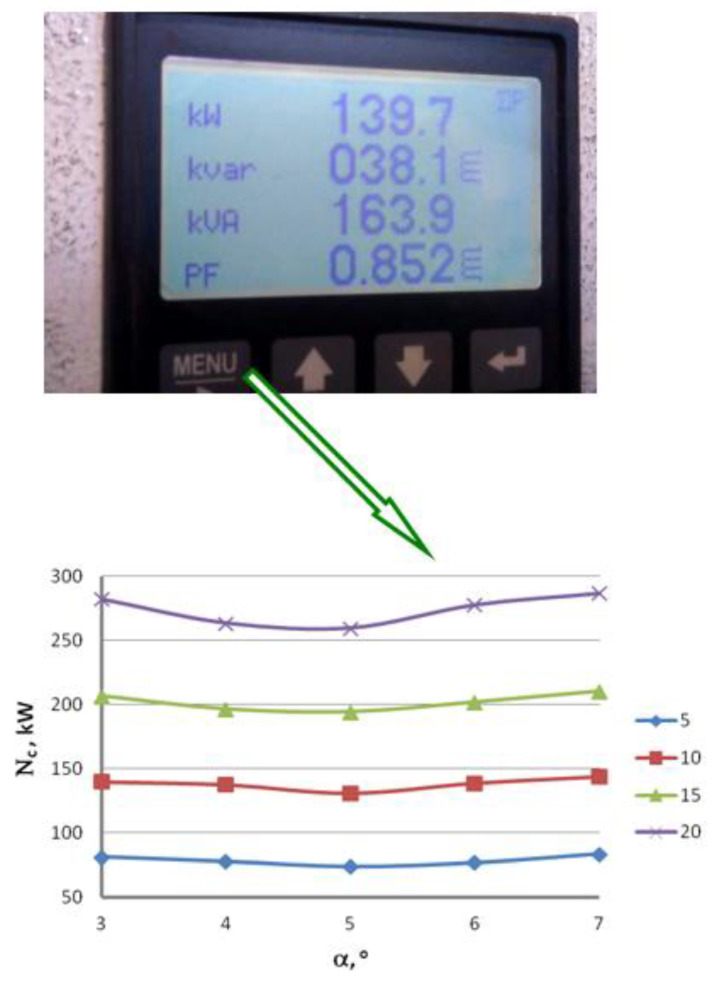
Power consumption reading field by the Mario Frigerio machine and the total drawing power as a function of a drawing angle for the wires drawn with a speed of v = 5, 10, 15 and 20 m/s.

**Figure 7:**
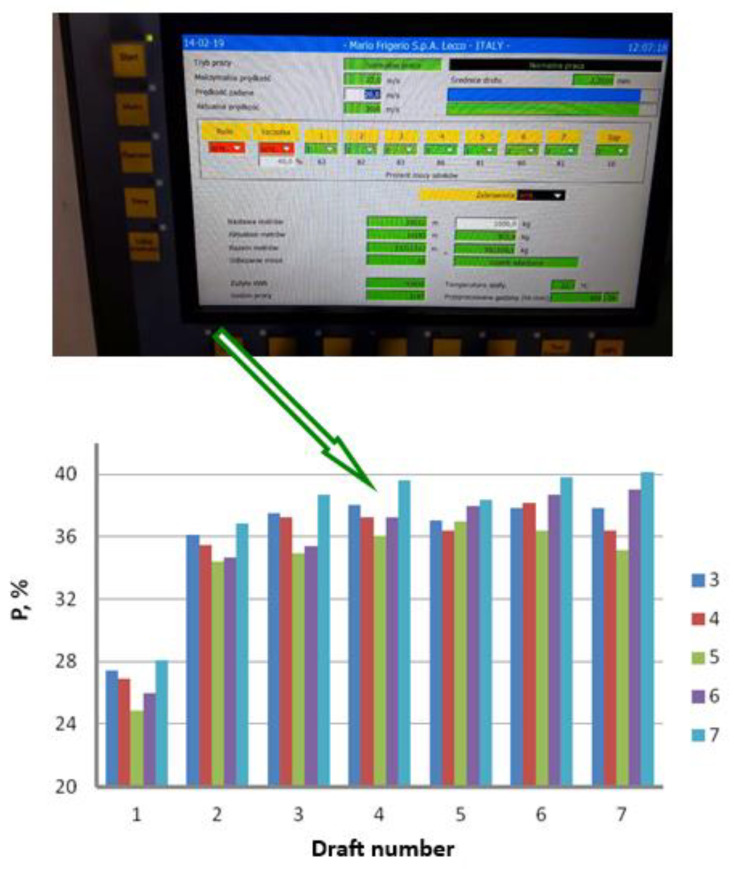
Control panel of the Mario Frigerio machine and percentage load of the engines P in respective drafts for the wires drawn with the angle α = 3, 4, 5, 6 and 7° and drawing speed of 20 m/s.

Experimental measurement of the drawing power was conducted in industrial conditions. In the first stage, we determined the total drawing power necessary to run the entire drawing process and then read the percentage load of the engines on the respective drawing stages from the control panel of the drawing machine. Figure 6 and Figure 7 demonstrate graphs showing the influence of the drawing technology on the actual force parameters of the drawing process.

The results of the industrial measurements of the drawing power, presented in Figure 6 and Figure 7, confirmed an essential influence of the die working angle on the force parameters of the drawing process, with the higher drawing speed exacerbating the impact of the α angle on the drawing process. The charts also show that the relation between the drawing power and drawing angle is not linear and achieves minimum for the 5° angle. Increasing or decreasing the angle translates to increasing the drawing power. The differences in drawing power between wires drawn in dies with an angle of α = 3° and α = 7°, depending on the drawing speed, range from 1.6 to 3%. The literature allows us to make an assumption [23] that there is a direct relationship between power and drawing tension. Thus, we can assume that lower drawing power corresponds to lower tension. Drawing tension value is a function of several components. The first one is the work related to the raw wire deformation, the second is the work of the friction powers, and the third is related to the occurrence of the shape deformations compounded with the macroshearing of the outer layers of the wire that additionally strengthens the latter. In addition, increasing the drawing angle reduces the contact surface between the wire and the die, which results in lower friction force and lower drawing tension. On the other hand, a higher α angle corresponds to higher deformation resistance and increased shape deformation, which results in increased drawing tension. It is corroborated by the experimental measurement of the drawing power, suggesting that either too small or too large an angle results in increased drawing tension. In the case of drawing the zinc-coated wires, as opposed to the drawing of uncoated wires, the determination of the optimal drawing angle is more complex. The friction between the wire and the die, inherent to the drawing process, results in the shearing of the zinc coating. Utilization of small drawing angles decreases the contact tensions and results in more fluent deformation of the zinc coating. It is worth remembering, however, that it also increases the friction surface, which translates to increased temperature on the wire surface. An increase in the drawing angle decreases the friction force in a die, which corresponds to slower heating of the wire, although the risk of shearing the zinc coating upon wire entry into the die increases. Due to all aforementioned factors, the authors of this paper posit that the optimal value of a drawing angle is α = 5°.

**Figure 8:**
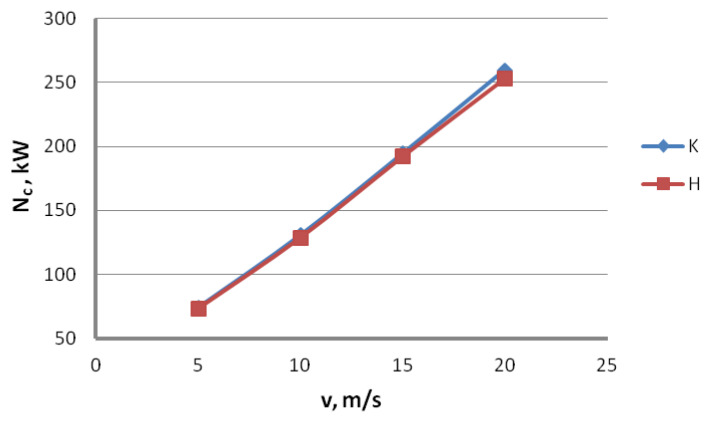
Total drawing power N_c_ as a function of the drawing speed for the wires manufactured using conventional K and hydrodynamic H methods.

**Figure 9:**
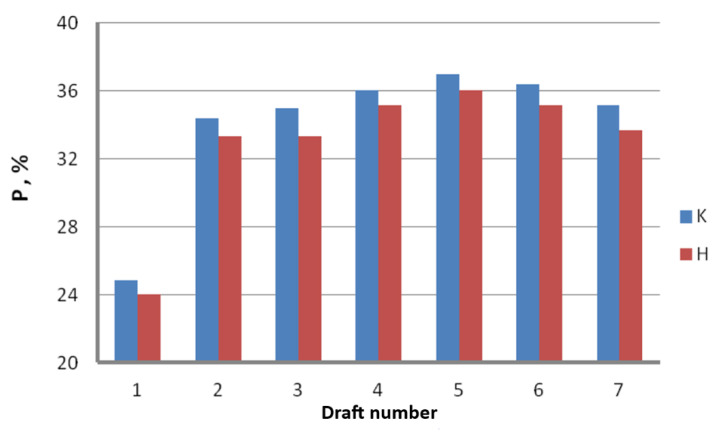
Percentage engine load P in respective drafts for the wires manufactured using conventional K and hydrodynamic H methods.

All the results of the theoretical drawing power calculations presented above suggest that it would be prudent to use hydrodynamic dies in drawing zinc-coated wires. This is why this paper compares the actual drawing power for the wires drawn using the conventional and hydrodynamic methods, as demonstrated in Figure 8 and Figure 9.

The research results presented in Figure 8 and Figure 9 have shown that the usage of hydrodynamic dies is beneficial for the power and force parameters of the zinc-coated wires drawing. In the entire analyzed range of drawing speed, the utilization of hydrodynamic dies resulted in a decrease in the drawing power. Depending on the drawing speed, the difference between the conventional and hydrodynamic methods ranges from 1.1% to 2.5%. The values of drawing power obtained through the experimental measurement are much lower than the values of theoretical calculations.

**Figure 10:**
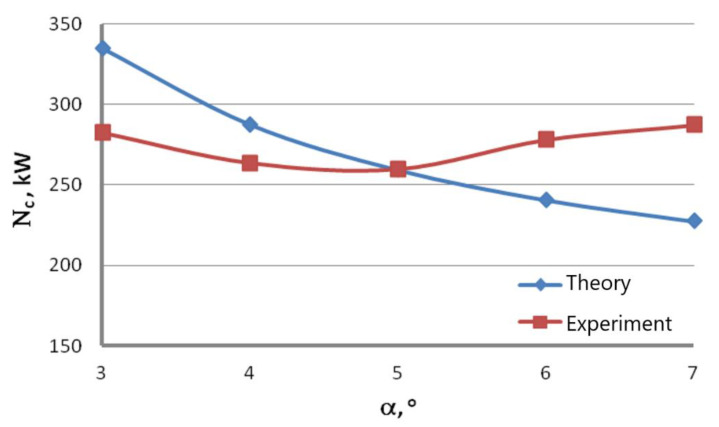
Change of the total drawing power, both theoretical and actual, as a function of the drawing angle for the wires drawn with the speed of 20 m/s.

**Figure 11:**
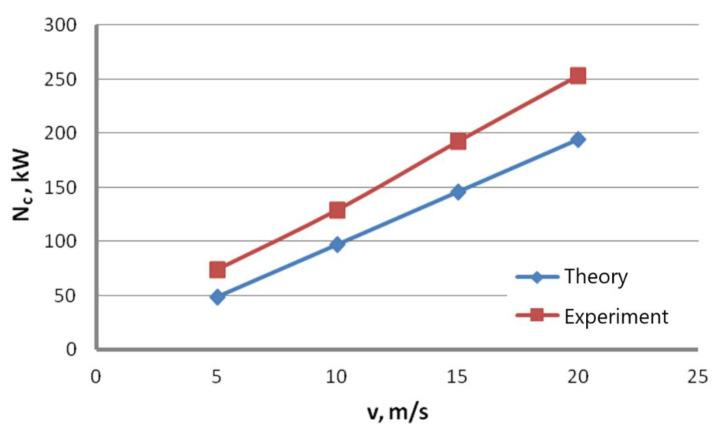
Change of the total theoretical and actual drawing power for hydrodynamic dies as a function of drawing speed.

To increase the quality of the drawing power analysis, the theoretical results were compared with the ones obtained experimentally. Figure 10 shows the change of the total drawing power, both theoretical and actual, as a function of the drawing angle for the wires drawn with the speed of 20 m/s, while Figure 11 shows the change of the total theoretical and actual drawing power for hydrodynamic dies as a function of drawing speed.

Data presented in Figure 10 and Figure 11 suggest that the equations available in the literature only allow for estimating the drawing power because they do not account for all factors influencing the drawing process. Calculations assume a constant value of the friction coefficient, even though, in practice, it differs between drafts and depends on the lubricating conditions and drawing speed. In addition, calculations did not account for the increasing shape deformations in wires drawn at high angles that cause additional deformation resistance and material hardening.

Thus, depending on the angle and drawing speed, the differences between the theoretical and experimental values of the drawing power can amount even to 20%. It has been demonstrated that Marcol’s equation [1] allows a relatively accurate estimation of force parameters for the wire drawing process in dies with an angle α = 5°, that, according to the authors of this paper, is optimal for drawing the zinc-coated wires. As for the hydrodynamic wire drawing, the differences in the drawing power between the conventional and hydrodynamic methods are significant and amount to, depending on the drawing speed, even 25%. The comparative analysis indicates that the friction coefficient value for the hydrodynamic dies in the real drawing process is much higher than 0.03.

## 4. Calculations for the Limitation of Energy Consumption, Material Losses and Pollution

**Figure 12:**
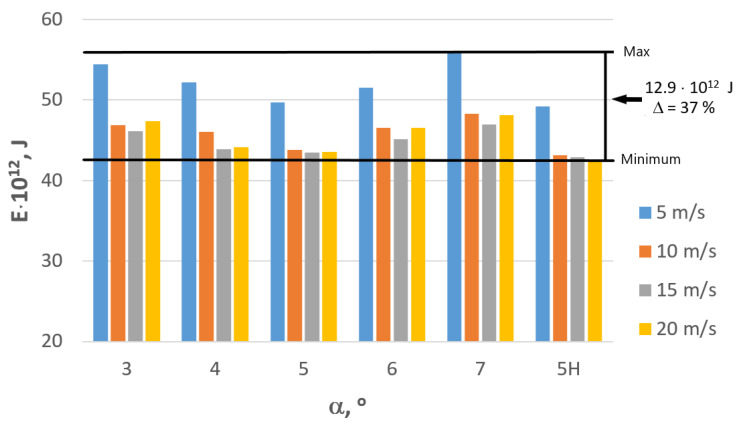
Influence of the technology of wire manufacturing on energy consumption in relation to the manufacturing of 100,000 tons of steel wire drawn with speed v = 5, 10, 15, 20 m/s.

Currently, millions of tons of zinc-coated wire are produced annually around the world. A large factory can manufacture approximately 100,000 tons of wire every year. Thus, for calculation purposes, the values related to consumption, losses and pollution correspond to the manufacturing of 100,000 tons of wire. Idemat software (app version 2.9.0.) has been used to analyze the influence of the consumption of electric energy produced by a coal-burning power plant on environmental pollution and associated eco-costs. Table 1 demonstrates the total instantaneous drawing power measured in the industrial conditions, while Table 2 and Figure 12 show the total amount of electric energy E required to manufacture 100,000 tons of 2.2 mm wire.

Using data presented in Table 2 and Figure 12, one can state that proper adjustment of the drawing process parameters, i.e., die geometry and drawing speed, allows for a substantial reduction of the energy consumption in the steel wire manufacturing process. The differences between analyzed variants amounted to approximately 37%, which in relation to an annual output of a wire factory translates to roughly 13 trillion Joules. It has been demonstrated that low drawing speed (close to 5 m/s) in the process of the zinc-coated steel wire drawing results not only in lower production efficiency but also in increased energy consumption, with the usage of high drawing angles exacerbating this phenomenon. 

**Figure 13:**
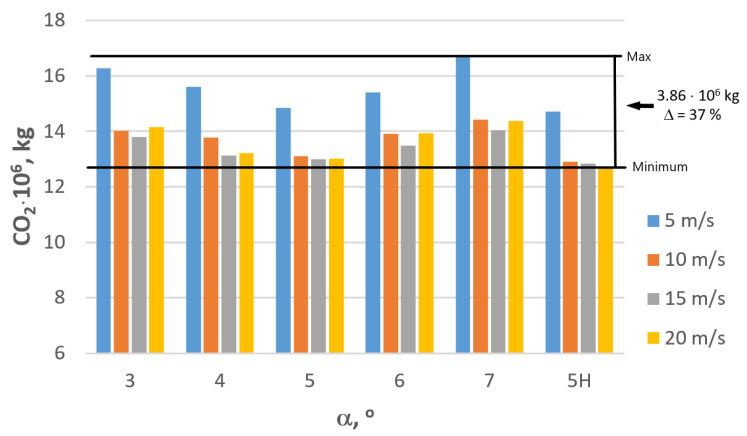
Influence of the technology of wire manufacturing on the CO_2_ emissions caused by energy consumption in relation to the production of 100,000 tons of steel wire drawn with v = speed 5, 10, 15, 20 m/s.

**Figure 14:**
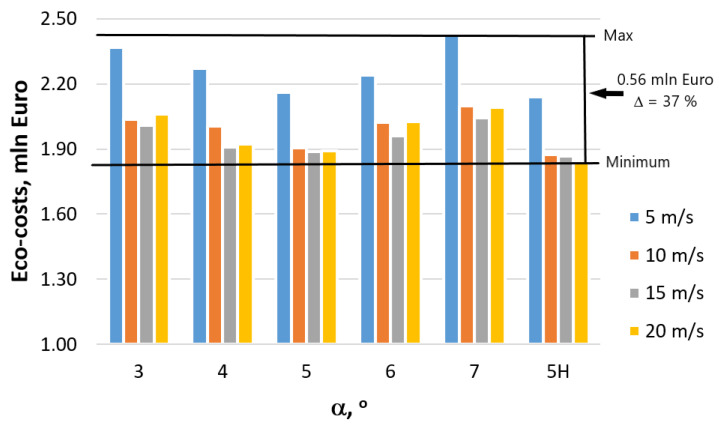
Influence of the technology of wire manufacturing on the CO_2_ emissions caused by energy consumption in relation to the production of 100,000 tons of steel wire drawn with speed v = 5, 10, 15, 20 m/s.

**Figure 15:**
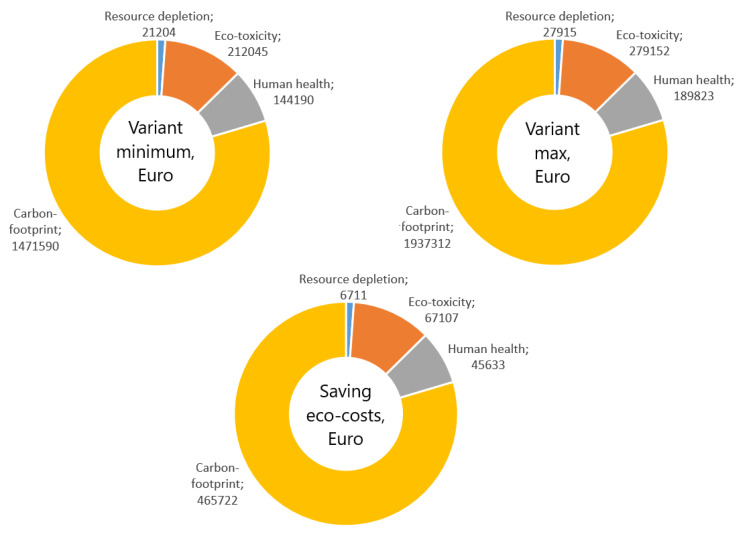
Influence of the technology of wire manufacturing on the generation of costs (in Euro) caused by energy consumption in relation to the production of 100,000 tons of steel wire.

Higher energy consumption translates to higher CO_2_ emissions. Table 3 and Figure 13, Figure 14 and Figure 15 demonstrate the influence of wire manufacturing technology on the CO_2_ emissions caused by electric power consumption.

Research results presented in Figure 13 clearly indicate that the factories manufacturing metal products have a lot of opportunities to reduce emissions. Modifying the technology based on the optimization of the die geometry and drawing speed allows for reducing the CO_2_ emissions by approximately 37%, which annually translates to almost 4000 tons of CO_2_, which more than justifies actions aimed at reducing energy consumption and environmental impact. Incentives to undertake such actions are steadily increasing environmental tariffs dependent on the annual production volume and manufactured inventory. Table 4, Figure 14 and Figure 15 present the influence manufacturing of wires has on the eco-costs incurred by the consumption of electric energy produced in coal power plants.

Analysis of eco-costs demonstrated that in the case of the annual steel wire manufacturing volume of 100,000 tons, the factory generates costs equal to EUR 2.5 mln, and implementation of proper technology allows for reducing eco-costs by almost EUR 0.5 mln. The eco-costs mentioned include resource depletion, eco-toxicity, human health, and carbon footprint. Thus, the data presented in this paper demonstrate how significant the impact of utilized technology is on the natural environment.

**Figure 16:**
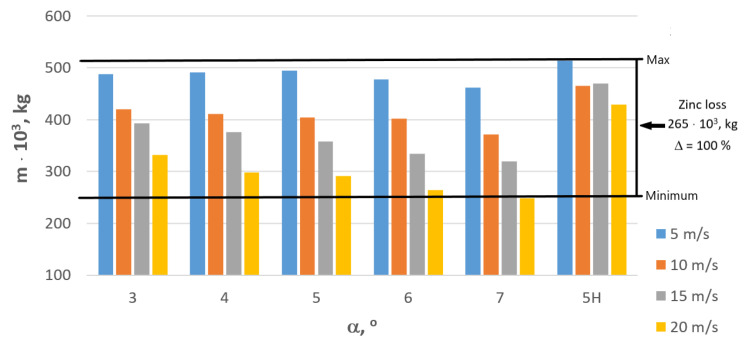
Influence of the technology of wire manufacturing on the mass of zinc coating in relation to 100,000 tons of steel wire drawn with speed v = 5, 10, 15, 20 m/s.

While analyzing the impact of the manufacturing processes on the natural environment, it is necessary to consider not only the energy consumption but also other components. In the case of zinc-coated steel wire, zinc-coating itself is one such component. Wires and wire products with zinc coating must be compliant with specified standards, including EN ISO 10244-2. This standard determines the wire class and corrosion resistance based on the wire diameter and coating thickness. When the zinc coating is too thin, the wire class is lowered. The wires are produced by pulling them through the dies, and the input material for such treatment is steel rolled after hot dip galvanization. While moving through subsequent dies, the zinc coating gets progressively thinner due to the mechanical elongation of the wire and shearing of the coating by the dies. The thickness of the zinc coating on the rolled steel depends on the required thickness of the coating on the final product. Thus, the higher are losses during the manufacturing process, the thicker the coating on the rolled steel. Table 5 and Figure 16 demonstrate the influence of the technology of wire manufacturing on the mass of the zinc coating per 100,000 tons of the zinc-coated steel wire.

**Figure 17:**
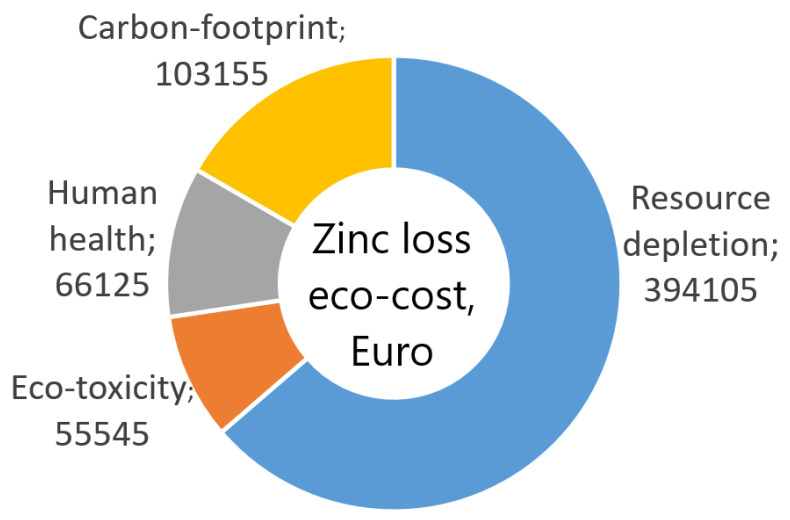
Costs generated by the loss of zinc in the production of 100,000 tons of steel wire.

Experimental research conducted in an industrial production facility demonstrated that the technology of wire manufacturing significantly influences the thickness of the zinc coating and the volume of lost zinc, with the differences between the analyzed variants amounting to 265 tons. This is corroborated by the results presented in Table 5, and Figure 16 which demonstrate a causal relationship between the increase in drawing speed and the angle of the contact zone of a drawing die and the increase of zinc loss, which also translates to increased CO_2_ emissions. According to Idemat, the production of 1 kg of pure zinc generates 3.33 kg of CO_2_. This, in turn, means that the annual production of wire referred to herein results in the emission of almost 900 tons of CO_2_; Figure 17.

The utilization of hydrodynamic dies that reduce friction between the material and tool in the wire manufacturing process had a beneficial influence on the coating deformation process, increasing the thickness of zinc coating by approximately 100%. This means that the demand for zinc in the zinc-coated wires manufacturing process was reduced by half, which translates to the reduction of all eco-costs related to zinc by approximately EUR 600,000. Zinc-coating of steel is one of the best-known toxic technological processes that significantly impact the environment because the total pollution includes the impact of zinc excavation and all metallurgical processes used to manufacture pure zinc utilized as a charge material for hot-dip coating lines. This type of coating requires the rolled steel to travel through the processing line, where steel is etched in acid baths (emission of gases and disposal of the used etching solutions), washed, and then coated by molten zinc in a galvanization bath (energy to melt zinc comes from electric or gas sources generating emissions of CO_2_ and other gases). Thus, the entire eco-costs related to the zinc-coating of steel are much higher than those shown in Table 5 and Figure 16. The data presented in this paper should encourage the manufacturers of zinc-coated steel products to modify current technologies of zinc-coating and steel processing.

## 5. Conclusions

Based on the theoretical and experimental research on the production of galvanized steel wires, the following conclusions can be drawn:

1. Empirical equations to analyze the energy consumption in the steel wire manufacturing process can be used only in a limited scope, as they do not address all the factors, causing discrepancies between theoretical and practical results.
2. Industrial measurements of the drawing power confirmed the significant influence of the angle of the drawing die contact zone on the force parameters of the drawing process, with the influence of the angle α on the drawing process increasing with the increase of the drawing speed.
3. Calculation of the energy consumption demonstrated that the utilization of the optimal wire drawing technology allows for a reduction of electric power by 37%, which translates to a reduction of annual consumption by 13 TJ, a reduction of annual CO_2_ emissions by 4000 tons and a decrease in total eco-costs by approximately EUR 0.5 mln.
4. Drawing technology influences the level of zinc coating loss and CO_2_ emissions. Proper parameters of the wire drawing technology allow for obtaining coating that is 100% thicker, which allows for saving 265 tons of zinc that would generate eco-costs of approximately EUR 0.6 mln.
5. Optimization of energy consumption and reduction of the greenhouse effect in the manufacturing processes is a complex issue, as it is usually a sum of several or more than a dozen separate manufacturing stages. The optimal parameters for the zinc-coated wires manufacturing process are hydrodynamic dies, drawing angle (angle of the working part of a die) α = 5°, and a drawing speed of 10–15 m/s.

## Figures and Tables

**Table 1:** Total drawing power N_c_ in the process of drawing wires with an initial diameter of 5.5 mm and end diameter of 2.2 mm, for wires drawn in conventional dies with a drawing angle α = 3, 4, 5, 6, 7°, and in hydrodynamic dies with a drawing angle α = 5° (5H), v—drawing speed.

v, m/s	Drawing Angle α, °					
	3	4	5	6	7	5 H
	Total Drawing Power N_c_, kW					
5	81.2	77.9	74.1	76.9	83.3	73.4
10	139.8	137.5	130.7	138.8	144.0	128.7
15	206.6	196.5	194.4	201.9	210.3	192.1
20	282.6	263.6	259.7	277.9	287.1	253.1

**Table 2:** The total amount of electric energy E required to manufacture 100,000 tons of 2.2 mm wire, depending on the drawing technology used, where α—drawing angle, H—hydrodynamic dies, v—drawing speed.

v, m/s	Drawing Angle α, °					
	3	4	5	6	7	5 H
	Electric Energy E · 10^12^, J					
5	54.42	52.21	49.66	51.54	55.83	49.20
10	46.85	46.08	43.80	46.51	48.26	43.13
15	46.16	43.90	43.43	45.11	46.98	42.92
20	47.35	44.17	43.51	46.56	48.11	42.41

**Table 3:** CO_2_ emissions related to the consumption of electric energy E required to manufacture 100,000 tons of 2.2 mm wire, depending on the drawing technology used, where α—drawing angle, H—hydrodynamic dies, v—drawing speed.

v, m/s	Drawing Angle α, °					
	3	4	5	6	7	5 H
	CO_2_ Emissions · 10^6^, kg					
5	16.28	15.62	14.85	15.42	16.70	14.71
10	14.01	13.78	13.10	13.91	14.43	12.90
15	13.81	13.13	12.99	13.49	14.05	12.84
20	14.16	13.21	13.02	13.93	14.39	12.68

**Table 4:** Eco-costs related to the consumption of electric energy E required to manufacture 100,000 tons of 2.2 mm wire, depending on the drawing technology used, where α—drawing angle, H—hydrodynamic dies, v—drawing speed.

v, m/s	Drawing Angle α, °					
	3	4	5	6	7	5 H
	Eco-Costs, EUR 1 mln					
5	2.37	2.27	2.16	2.24	2.43	2.14
10	2.04	2.00	1.91	2.02	2.10	1.88
15	2.01	1.91	1.89	1.96	2.04	1.87
20	2.06	1.92	1.89	2.03	2.09	1.84

**Table 5:** Total mass of zinc per 100,000 tons of 2.2 mm wire, depending on the drawing technology used, where α—drawing angle, H—hydrodynamic dies, v—drawing speed.

v, m/s	Drawing Angle α, °					
	3	4	5	6	7	5 H
	Zinc Mass m·10^3^, kg					
5	487.83	490.82	494.5	477.25	462.07	513.36
10	420.21	411.24	404.57	402.04	371.22	464.83
15	393.53	375.82	358.34	333.96	320.16	469.43
20	331.89	298.08	291.41	264.73	248.86	428.72

## Data Availability

Not applicable.
